# Experience in Rehabilitation Medicine Affects Prognosis and End-of-Life Decision-Making of Neurologists: A Case-Based Survey

**DOI:** 10.1007/s12028-018-0661-2

**Published:** 2019-01-03

**Authors:** Annette Rogge, Victoria Dorothea Witt, José Manuel Valdueza, Christoph Borzikowsky, Alena Buyx

**Affiliations:** 1 Institute for Experimental Medicine, Medical Ethics, Kiel University, University Hospital Schleswig-Holstein, Arnold-Heller-Str. 3, Haus 28 (Gebäude Rechtsmedizin), 24105 Kiel, Germany; 2 Neurological Center, Segeberger Kliniken, Hamdorfer Weg 3, 23795 Bad Segeberg, Germany; 3 Institute for Medical Informatics and Statistics, Kiel University, University Hospital Schleswig-Holstein, Brunswiker Straße 10, Haus 31, 24105 Kiel, Germany; 4 Institute for History and Ethics in Medicine, Technical University Munich, Munich, Germany

**Keywords:** Life-sustaining therapy, Neurointensive care, Prognosis, End-of-life decision, Disorder of consciousness, Stroke

## Abstract

**Background:**

Outcome predictions in patients with acute severe neurologic disorders are difficult and influenced by multiple factors. Since the decision for and the extent of life-sustaining therapies are based on the estimated prognosis, it is vital to understand which factors influence such estimates. This study examined whether previous professional experience with rehabilitation medicine influences physician decision-making.

**Methods:**

A case vignette presenting a typical patient with an extensive brain stem infarction was developed and distributed online to clinical neurologists. Questions focused on prognosis, interpretation of an advanced directive, whether to withdraw life-sustaining treatments and information on prior rehabilitation experience from the survey respondent.

**Results:**

Of the participating neurologists, 77% opted for the withdrawal of life-sustaining therapies (*n* = 70; response rate: 14.8%). This decision was not affected by age, gender, or length of clinical experience. Neurologists with experience in rehabilitation medicine tended to estimate a more positive prognosis than neurologists without, but this result was not significant (*p* = .13). There was an association between the intervention chosen and previous experience in rehabilitation; neurologists with experience in rehabilitation medicine opted significantly more often (31.8%) for continuing life-sustaining treatments than neurologists without such experience (8.7%, *p* = .04).

**Conclusion:**

Our results indicate that there are subjective factors influencing decisions to limit life-sustaining treatments that are based on previous professional experience. This finding emphasizes the variability and cognitive bias of such decision processes and should be integrated into future guidelines for specialist training on end-of-life decision-making.

## Introduction

With increasing therapeutic possibilities in intensive care, questions about prognostic evaluation, treatment goals, and decision-making on limiting life-sustaining treatments are becoming increasingly important and even more complex.

In particular, disorders of consciousness and severe neurologic injuries, such as large brainstem stroke, challenge both caregivers and family members when deciding whether there is a chance of meaningful recovery, and based on that assessment, whether it would be in the patient’s interest to go through the act of withdrawing life support [1–4].

There is a great variety of patient outcomes, as well as differences in patient’s psychological and emotional responses to these outcomes [5, 6]. In addition, different international studies have shown that the accuracy of the estimated prognosis of patients with neurologic conditions is limited [7, 8]. Although there have been promising efforts to create simple index scores to predict outcomes [9], there are still too many differences in derivation and validation techniques as well as practical implementation methods that limit their use [10, 11]. Currently, no fully objective, reliable scoring system or decision algorithm exists, which means that the neurologist/neurointensivist makes the final decision regarding prognosis [12]. Even while human clinical experience can still beat scoring systems [13], the “human factor” in decision-making comes with a number of challenges, including a “uniformed summary judgement based on faulty pattern recognition, inadequate outcome data, sole reliance on retrospective studies, statistical limitations, nongeneralizability of outcome data, or the fallacy of the self-fulfilling prophecy” [14, p. 107].

Objective clinical evidence in acute stroke that impacts end-of-life decisions includes for instance disturbance of consciousness at presentation, dysphagia in an assessment of dysphagia, swallowing and speech on day 1 or a large supratentorial stroke [15]. Relevant technical findings are bedside monitoring, clinical examinations, and imaging [8]. However, it has been highlighted that the quality of evidence available for the assessment of outcome prediction in patients with severe stroke is often less than optimal overall and must be interpreted with caution [16].

The “human factor” in estimating clinical outcomes for such patients is not yet fully understood. Without a doubt, emotive and subjective factors play a role [17]; apparently, even clinicians themselves believe that their ability to prognosticate is not a matter based on the fact [8]. Nevertheless, prognostication has a very strong impact. If a physician’s estimate of intensive care unit (ICU) survival is less than 10%, this is strongly associated with treatment withdrawal and predicts mortality more powerfully than illness severity, evolving or resolving organ dysfunction, and use of inotropes or vasopressors [18]. A number of studies have shown that age, gender, religion, and geographic region are associated with willingness to forgo life-sustaining treatments [19–21]. The influence of geographic region is likely because attitudes and legal settings regarding end-of-life decision-making vary within Europe [22, 23].

Beyond age, sex, religion, and region, which factors influence physicians’ process of determining prognosis? Drawing on our daily praxis working in rehabilitation medicine and ethics consults, we asked if previous experiences with clinical outcomes of stroke patients might influence the decision-making in the intensive care setting. Physicians with experience in rehabilitation medicine are more likely to have seen improvement in the functional performance of patients with disorders of consciousness [24], as they follow the patient for a longer time. Therefore, they might be more optimistic and prefer to opt for life-sustaining therapies.

## Study Aims

The aim of the present study was to investigate the influence of neurologists’ previous professional experience on decision pathways in end-of-life decision-making in stroke cases using a fictional vignette case. The participants were blinded to our research questions:

- (A) Are neurologists with experience in rehabilitation medicine more likely to estimate a more positive prognosis than those without such experience?
- (B) Are neurologists with experience in rehabilitation medicine more likely than neurologists without such experience to opt for continuation of life-sustaining therapies?
- (C) Is there an association between estimated prognosis, respective confidence in that estimation, and (a) gender, (b) age, or (c) professional experience of the treating physicians?

## Methods

### Study Design and Measurements

**Fig. 1:**
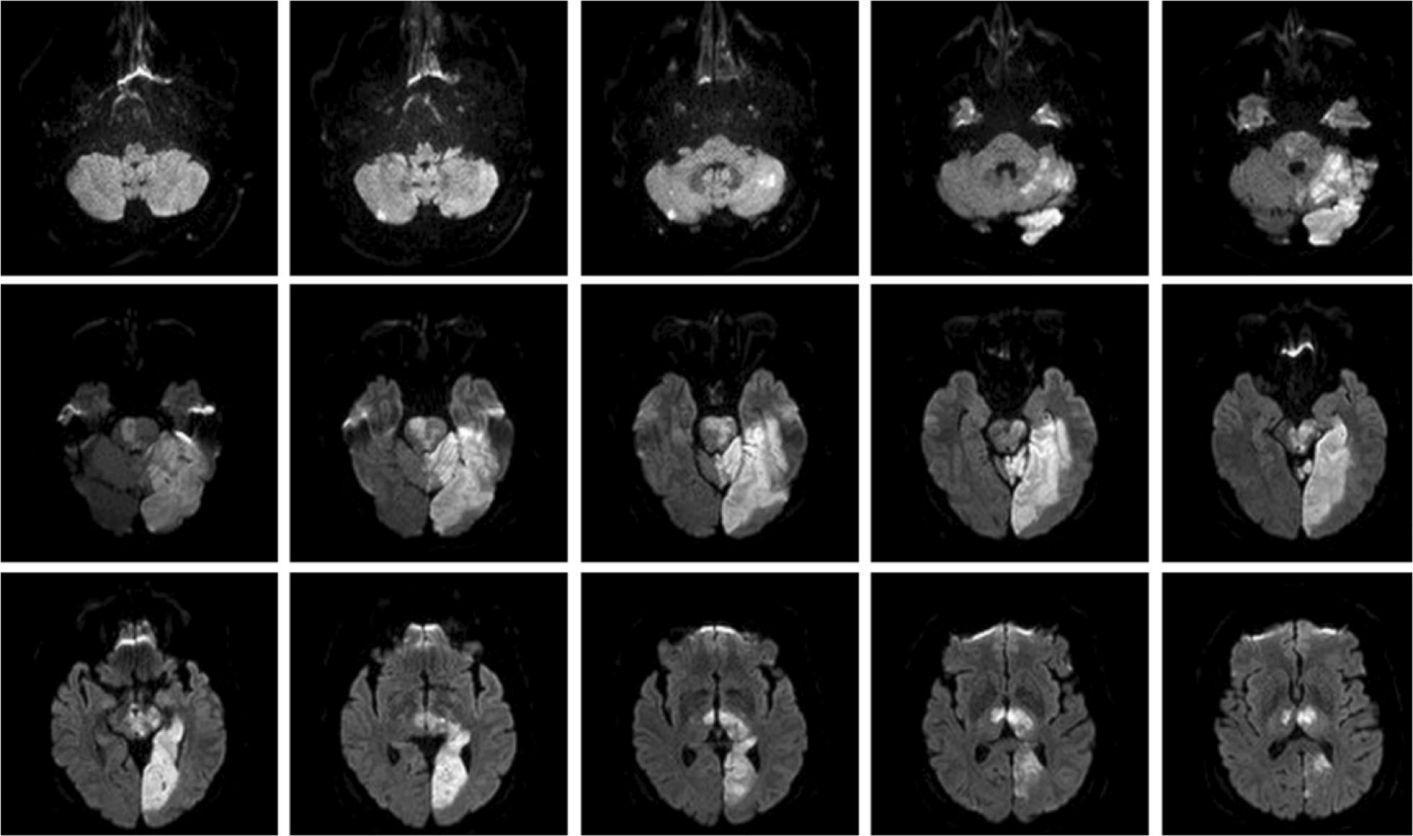
MRI scan of fictional patient Mr. M. presenting defects following a basilar artery embolism as part of a case vignette aimed at neurologists

The online questionnaire (https://medizinethikerin.de/docs/English_Version_of_questionnaire.pdf) was designed by a trained neurologist with advanced training in medical ethics (AR) and was further developed in close interdisciplinary collaboration of the coauthors (specializing in ethics, neurology, and statistics). The questions were aimed at neurologic specialists working in stroke units or in the ICU. Clinicians were given a fictional case vignette that was developed from a typical magnetic resonance imaging (MRI) image of a basilar artery embolism, with a step-by-step presentation of further clinical and technical findings. The image as shown in Fig. 1 was described as a day 7 MRI of a 75-year-old, continuously unconscious patient (“Mr. M.”) with bilateral mesencephalic, cerebellar and thalamic infarctions, right-sided pons infarction, and a left-sided posterior infarction while the basilar artery was recanalized.

In a next step, the main excerpts of an advanced directive signed by the fictional patient based on a template drafted for the general public by the German Federal Ministry of Justice and Consumer Protection were offered. The advance directive was written to indicate that the patient would be in favor of withdrawing or withholding life-sustaining if the prognosis for regaining consciousness and communicative abilities was poor. A wife with full power of attorney was introduced. The wife was described as being undecided regarding limitation of treatment and open to suggestions from the clinician.

Questions focused on the typical decisions that would have to be made at that time of the clinical pathway (day 10 after stroke) with substantial implications for the long-term therapy goal, specifically, a feeding tube and tracheostomy. Participants were asked for their estimate of the prognosis of the patient and their opinion concerning further life-sustaining therapies. With regard to the advance directive, they were asked to decide whether a feeding tube and tracheostomy would correspond to the patient’s wishes in the given situation. Finally, they were asked how often in their daily practice they came across these or similar questions regarding life-sustaining therapy decisions within a year. Table 1 provides an overview of the five main items that the online study addressed with corresponding answer options.

**Table 1:** Overview of the five main items and corresponding answer options of the questionnaire

Question	Answer options
1: I consider the probability to regain consciousness and ability to communicate for this patient as…	Unlikely (1) → Likely (10) (rated between 1–10 on Likert-like scale)
2: How confident are you in your prognostic estimate?	Very uncertain (1) → Very certain (10) (rated between 1–10 on Likert-like scale)
3: Based on the offered advance directive, I would suggest that insertion of a feeding tube and tracheostomy corresponding to the will of Mr. M.	Correct or incorrect
4: I would argue for the execution of the above procedures	Yes or no
5: How often do you come across these or similar questions on life-sustaining therapies during your professional daily routine?	Never, 1–2x/year, 5–10x/year, more often than that

Other items of the questionnaire were concerned with personal data and work experience characteristics. These comprised questions on age, gender, medical specialization(s), current task area (e.g., ICU, stroke unit), length of experience in acute medicine, length of experience in rehabilitation medicine, and the first two postcode numbers of the respondent’s current location in Germany. Experience in rehabilitation medicine was defined as having worked in a specialized neurologic rehabilitation ward or hospital. The German healthcare system clearly differentiates between acute and rehabilitation medicine. We also asked for the years the colleagues have been worked in rehabilitation medicine (none/< 2 years/2–5 years/5–9 years/more than 10 years).

Questionnaires were included in the analysis as long as all key questions specified in Table 1 were answered, even if participants declined to answer individual questions regarding their personal background.

The questionnaire was pretested and modified by three different, experienced neurologists who did not participate in the study. It was evaluated by the research ethics committee at University of Kiel; ethics approval (No. D407/17) was issued prior to the start of the study.

### Methods

Data were collected online with the evaluation- and survey software *Evasys Survey Automation Suite* (Version 7.0, Electric Paper Ltd., 2016). Specialist status in neurology and employment on an ICU or stroke unit were the main inclusion criteria. All hospitals with certified stroke units listed by the German Stroke Society and the Internet presence were contacted to identify appropriate respondents, who were then invited via email to participate and to give their informed consent for participating and publishing of study results. Participation was voluntary, and no incentives were offered. The initial contact email included the aim, objectives, and basic content of the study without any references to the underlying hypotheses. Voluntariness and data protection rules were explained. All data were gathered anonymously. To prevent multiple survey completion, the online link to the Web site was protected by a transaction authentication number that expired after single participation. If the invited neurologists did not respond, up to four reminders were sent via email. Invalid email addresses were excluded. Data were gathered between February 2 and April 7, 2017.

### Sample

A total of 499 neurologists were invited. In total, *n* = 74 neurologists participated (response rate: 14.8%), and four had to be excluded from the analysis because one lacked a prognosis estimate, one lacked specialist status as defined by the German Medical Association, and the remaining two had not consented to publishing. Therefore, *n* = 70 questionnaires were fully completed regarding the central questions (see Table 1). In individual cases, if answers to personal data were declined, the records were still included when all central questions of the questionnaire were completed.

### Statistical Analysis

Data were analyzed with *IBM SPSS Statistics* for Windows (Version 22.0.0.2, IBM, 2013). A nonparametric Whitney–Mann U test was performed to compare ratings between neurologists with and without rehabilitation medicine experience regarding the first two items of the questionnaire (Question 1 and 2, see Table 1). Differences in frequencies on life-sustaining therapies (Question 4, see Table 1) between neurologists with and without rehabilitation medicine experience were statistically analyzed with a *χ*^2^-test. For dichotomous variables (e.g., gender), we applied Spearman’s rank correlation coefficient. Pearson’s correlation coefficient was used for all other variables with continuous scales. A *p* value < .05 was considered statistically significant.

## Results

### Participants

The mean age was 45.9 years (standard deviation [SD] 5.8) which corresponds with the peak in age distribution of 40–49 years in German neurologists (data for mean age not available). The majority of our participants (76%) were male and deviated slightly from the gender ratio of neurologists across Germany (55% males, Table 2) [25]. All participants had experience in acute medicine, and many of them had 10 years or more (83%). Two-thirds (66%) also had experience in rehabilitation medicine (*n* = 3 n/a). Demographics and professional experience are further specified in Table 2.

**Table 2:** Characteristics of participants

	All participants (*n* = 70; *n* = 3 N/A* regarding prior experience)	Participants without prior experience in rehabilitation medicine (*n* = 23)	Participants with prior experience in rehabilitation medicine (*n* = 44)
Age (years), median	46 (SD 5.8)	45 (SD 6.3)	47 (SD 5.5)
Gender, *n* (%) (*n* = 1 N/A*)			1(2) N/A*
Female	16 (23)	6 (26)	8 (18)
Male	53 (76)	17 (74)	35 (80)
Primary discipline neurology, *n* (%)	70 (100)	23 (100)	44 (100)
Experience in acute medicine, *n* (%)			
None	0	0	0
< 2 years	1 (1)	1 (4)	0
2–5 years	3 (4)	1 (4)	2 (5)
5–9 years	8 (11)	0	7 (16)
10 and more years	58 (83)	21 (91)	35 (80)
Experience in rehabilitation medicine *n* (%) (*n* = 3 N/A*)			
None	23 (34)	23 (100)	0
< 2 years	11 (16)		11 (16)
2–5 years	18 (27)		18 (27)
5–9 years	8 (12)		8 (12)
10 or more years	7 (10)		7 (10)
Additional qualification in palliative medicine *n* (%)	3 (4)	1 (4)	2 (5)
Additional qualification in geriatric medicine *n* (%)	8 (11)		8 (18)

### Neurologists’ Prognosis and Confidence in Their Estimate

**Fig. 2:**
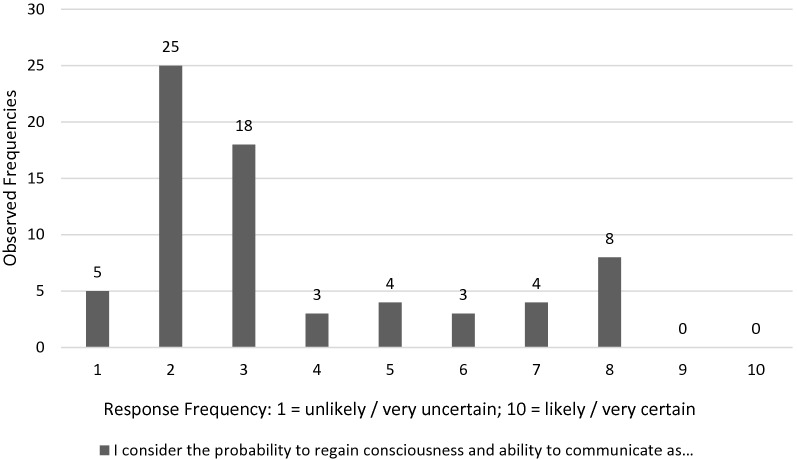
Neurologists individual response frequencies on probability to regain consciousness and ability to communicate (Question 1, see Table 1) on a Likert like scale from 1 = *unlikely/very uncertain* to 10 = *likely/very certain*

**Fig. 3:**
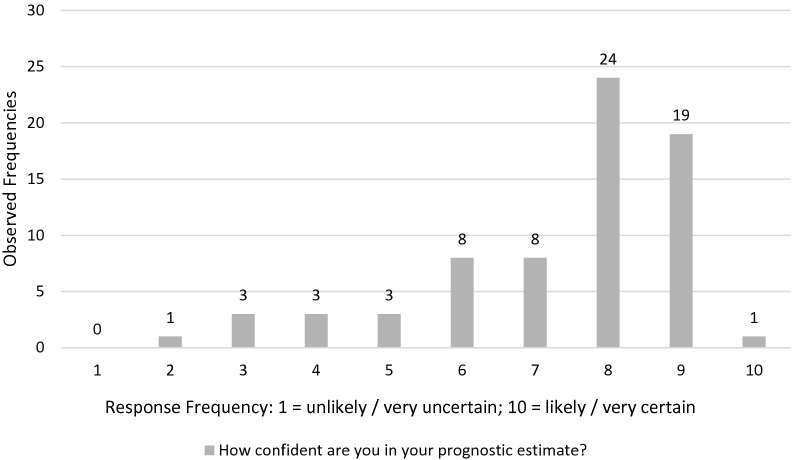
Neurologists individual response frequencies on confidence in their prognostic estimate (Question 2, see Table 1) on a Likert like scale from 1 = *unlikely/very uncertain* to 10 = *likely/very certain*

When asked how likely respondents considered the probability for the patient to regain consciousness and ability to communicate (see Question 1 in Table 1, Fig. 2), the majority decided within the spectrum of *unlikely* (mean 3.6, SD 2.2, range 1–8). Simultaneously, the majority of participants ranked their confidence regarding this estimate on the *certain* side of the spectrum (mean 7.4, SD 1.79, range 2–10, see Fig. 3).

The correlation between Question 1 and Question 2 was significantly negative, *r*(70) = − .30, *p* = .01.

### Zero-Order Correlations Between Age, Gender, and Years of Experience

There were no significant correlations between Question 1 (see Table 1) and gender (*r*(69) = − .04, *p* = .73), age (*r*(66) = .06, *p* = .64), or years of experience (*r*(70) = .11, *p* = .37). There were also no significant correlations between Question 2 (see Table 1) and gender (*r*(69) = .07, *p* = .59), age (*r*(66) = − .02, *p* = .89), or years of experience (*r*(70) = − .12, *p* = .32).

### Decision for Life-Sustaining Therapies

Based on the advance directive provided, the majority of neurologists assumed that insertion of a feeding tube and tracheostomy would not correspond with the will of the fictive patient, Mr. M. (Question 3, *n* = 56, 80%). The majority of clinicians would not argue for the execution of the procedures in this case (Question 4, *n* = 54, 77%).

When relating Question 1 to Question 4 (see Table 1), there were three participants (4% of *n* = 70) who, despite their positive prognosis (as defined by ratings of 6–10 on the Likert-like scale) for the patient, opted against proceeding with life-sustaining therapies, while four participants (6%) opted for proceeding with life-sustaining therapies despite their poor prognosis for the patient (as defined by ratings of 1–5 on the Likert-like scale). Reviewing relations between Questions 3 and 4 (see Table 1), three participants (4%) concluded from the advance directive that the patient would wish for life-sustaining therapies but opted against execution of the procedures. Five participants (7%) who interpreted the advance directive as such that the patient would not wish for life-sustaining therapies argued for executing the procedures.

Regarding how often participants would come across these or similar questions on life-sustaining therapies (Question 5) during their professional daily routine, a very small minority replied “never” (*n* = 1, 1%) and a majority “more often than 10 times a year” (*n* = 44, 63%); with almost ten percent answering “1–2 times a year” (*n* = 6, 9%), and roughly a third “5–10 times a year” (*n* = 19, 27%).

### Experience in Rehabilitation Medicine

**Fig. 4:**
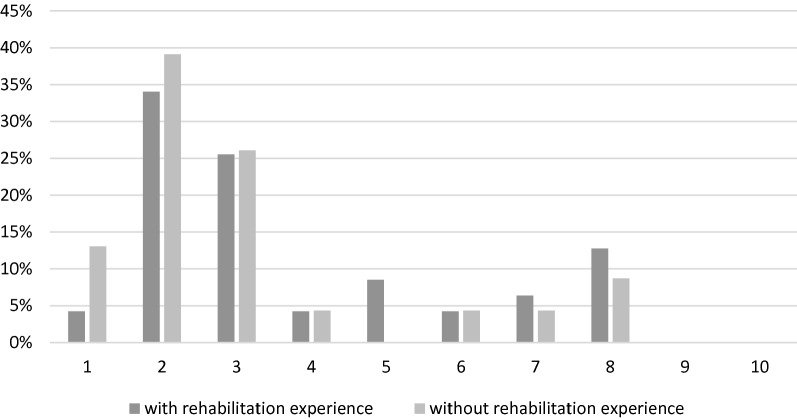
Comparison of estimated prognosis in neurologists with vs. without rehabilitation experience

The results shown in Fig. 4 were analyzed separately for neurologists with and without rehabilitation experience in relation to their answers on prognosis. There was no significant difference between the two groups regarding prognosis (*U*(23,44) = 393.5, *p* = .13), but the data show a nonsignificant trend toward more optimistic prognosis among those with rehabilitation experience.

Regarding decision-making, in total 32% (*n* = 14 of all *n* = 44) neurologists with experience in rehabilitation medicine would decide to proceed life-sustaining therapies, whereas 68% (*n* = 30) were against them. In the group with only acute medicine experience (*n* = 23), 2 neurologists (9%) decided to proceed with life-sustaining therapies and 21 (91%) opted against them. To compare the decisions on life-sustaining therapies of the two groups, a *χ*^2^ test was conducted to look at the distribution of the observed frequencies. In this case, neurologists without rehabilitation experience opted significantly more often against life-sustaining therapies (*χ*^2^(1, *n* = 67) = 4.44, *p* = .04).

## Discussion

We analyzed neurologists’ estimated prognosis of a fictional stroke patient and their subsequent decisions regarding life-sustaining interventions. The aim was to test the hypothesis that neurologists with experience in rehabilitation medicine would estimate a more positive prognosis and opt for the continuation of life-sustaining therapies. Previous studies have shown that physicians’ personal characteristics such as age, gender, religion, or geographic location play a role in estimating prognoses and (end-of-life-) decision-making [2–4, 8, 19–21, 26–30]. Thus far, the role of previous experience in rehabilitation medicine has not been investigated.

Overall, participating neurologists were rather pessimistic regarding the prognosis of the patient presented in the case vignette with regard to regaining consciousness and communicative abilities, with a statistically nonsignificant trend of those with rehabilitation medicine experience giving a more positive estimate.

Although there was a rather “pessimistic” view on the likelihood of regaining consciousness when reviewing the answers in our study, neurologists showed a large range of opinions (see Fig. 2), which demonstrates the high variability of neurologists’ estimates. This finding is in accordance with results from a pediatric study which investigated pediatric end-of-life decisions based on case scenarios. Randolph et al. also found a high interpersonal variability for the level of care physicians would suggest in these cases [27].

Despite the high variability found in our study, the confidence of the participants in their own estimation was high (Fig. 3), which once more affirms the importance of steps toward enhancing awareness of any sort of variability, acknowledgement of contributing factors and efforts to increase the consistency of communicating prognosis in neurocritical care [26].

In contrast to previous studies [19], in our sample, there was no significant correlation between gender, age or years of experience, and the estimation of the prognosis or neurologists’ confidence in their prognosis. This finding might be a coincidence or a problem with our small sample size.

The majority of our participants thought that a feeding tube and tracheostomy would not correspond to the will of the patient and opted against the life-sustaining therapies. Interestingly, three participants subsequently opted against the life-sustaining therapies, although they believed (albeit perhaps mistakenly) that the patient would wish for them. Five participants thought that the patient would not wish for the interventions, yet nonetheless opted for performing the procedures. If these replies were not mistakenly made, this result would be in line with experiences that show that patient will is sometimes ignored or even thwarted [31–33]. As we did not ask for explanations of individual decisions, further interpretation of this point is limited but definitely worth further investigation.

In our sample, the neurologists with experience in rehabilitation interpreted the case vignette differently, or at least derived different conclusions given that fewer of them would withhold life-sustaining procedures compared to neurologists without experience in rehabilitation medicine (68% vs. 91%). The assumption that prognosis is an important decision-making factor is supported by the fact that a majority of participating physicians gave a poor prognosis, and a majority would not argue for the life-sustaining procedures. Nonetheless, there was a minority who opted for proceeding with life-sustaining therapies despite their negative prognosis for the patient (6%, *n* = 4) or against life-sustaining therapies despite their positive prognosis for the patient (4%, *n* = 3). The study design does not allow for further conclusions at that point as we did not ask for explanations for decision-making.

According to our study, results there was no significant difference in prognostic estimation between neurologists with experience in rehabilitation medicine and those who had none (see Fig. 4). However, regarding decision-making, neurologists with previous experience in rehabilitation medicine were significantly more likely to opt for life-sustaining therapies. Thus, our data support the hypothesis that there are significant differences in end-of-life decision-making processes relating to previous rehabilitation experience.

One potential reason for these findings that have been discussed but not tested is that acute and rehabilitative medicine occurs in different departments and institutional settings. Therefore, intensive care physicians might lack the opportunity to follow-up with their patients after hospital discharge, leading to lower estimates of future quality of life and a stronger inclination to terminate life-sustaining treatments [34]. In any case, the following conclusions can be drawn from our findings. First, there are factors influencing physicians’ prognoses, and in particular, there are factors surrounding decisions about life-sustaining interventions that lie within the individual physician—such as types of previous professional experience. Second, it would likely be helpful to bring the two fields that are involved in the care of patients with severe stroke and other disorders of consciousness—acute/ICU care and rehabilitation medicine—closer together to reduce biases on both sides.

Families and surrogate decision makers regularly feel left out of the end-of-life decision-making process [35] and on occasion have difficulties trusting physicians [36]. Although neurologists in our study were overall confident in their prognoses, it should be noted how crucial it is to understand the limitations of prognostic information as physicians, and the factors influencing end-of-life decisions in the ICU, in order to ensure honest and effective communication with families [36, 37]. Such challenging decisions for patients with disturbances of consciousness always include the anxiety of possibly missing a “window of opportunity” [38] especially for the surrogate decision makers. Estimating the width of this window provides the basis for the treating physician’s suggestions and therefore it is fundamental to identify the factors influencing in this process [39, 40].

### Limitations

A case vignette approach always describes one of a potentially indefinite number of scenarios and variations. However, as described above, in the past, a case vignette approach has been used successfully in a multitude of studies; it is an established and validated tool [41]. Although we are confident that the case elicited reliable views of the study participants, testing more cases in future research could further strengthen the results of the present study. Other potential influencing factors such as cultural or religious beliefs were not investigated in this study. Another limitation is the low participation rate, which is not unusual for this type of qualitative, online-based research [42, 43]. Therefore, our results may not be representative and have to be interpreted cautiously. Finally, our results should be compared to those of other countries to examine cultural differences in the interpretation of informed consent and legal frameworks [22].

## Conclusion

There was a significant association in the analyzed sample of neurologists between their previous professional experience (in rehabilitation medicine) and their therapeutic decisions in a stroke patient. The results indicate that previous professional experience is one of the individual factors influencing decisions on therapy limitations beyond objective scales and technical findings. This emphasizes the known variability of prognosis and decision processes and is something that decision makers should be aware of. Furthermore, the results of this study suggest steps to raise awareness of individual influencing factors, and new consideration of whether a closer integration of acute and rehabilitation medicine in neurology would be beneficial in the future.
